# Propofol specifically suppresses IL-1β secretion but increases bacterial survival in *Staphylococcus aureus*-infected RAW264.7 cells

**DOI:** 10.1007/s11010-018-3348-2

**Published:** 2018-04-17

**Authors:** Ming-Shan Chen, Wen-Chun Lin, Hsuan-Te Yeh, Chia-Lin Hu, Shew-Meei Sheu

**Affiliations:** 1 Department of Anesthesiology, Ditmanson Medical Foundation Chia-Yi Christian Hospital, Chia-Yi, Taiwan; 2 Department of Medical Research, Ditmanson Medical Foundation Chia-Yi Christian Hospital, Chia-Yi, Taiwan; 3 Department of Medical Laboratory Science and Biotechnology, Chung Hwa University of Medical Technology, Tainan, Taiwan

**Keywords:** Intravenous anesthetics, Propofol, *S. aureus*, IL-1β, ROS

## Abstract

Anesthetics have immunomodulatory effects, but the use of different assay systems has contributed to inconsistent results in the literature. IL-1β and reactive oxygen species (ROS) secreted by phagocytes are important factors that protect against *Staphylococcus aureus* infection. In this study, the effects of four intravenous anesthetics (propofol, thiamylal sodium, midazolam, and ketamine) on IL-1β secretion, ROS, and bacterial survival in *S. aureus*-infected RAW264.7 cells were evaluated. *S. aureus*-infected RAW264.7 cells with or without intravenous anesthetic treatment were established as the experimental model. Cell supernatants were subjected to ELISAs to measure secreted IL-1β. Cell pellets were subjected to qPCR and western blot analyses to analyze IL-1β mRNA and protein levels. Luminol chemiluminescence assays were used to detect ROS, and bacterial survival was determined by counting the colony forming units at the beginning and end of the infection. Compared with the levels after treatment with the other intravenous anesthetics, secreted IL-1β levels were lowest in the supernatant of *S. aureus*-infected RAW264.7 cell cultures after propofol treatment, but propofol did not decrease IL-1β mRNA or protein expression. However, thiamylal sodium and midazolam decreased IL-1β mRNA and protein expression in a dose-dependent manner. Additionally, propofol substantially decreased *S. aureus*-stimulated ROS and phagocytosis. Bacterial survival was strongly increased by propofol treatment. Of the four intravenous anesthetics, propofol was the most potent inhibitor of IL-1β secretion and ROS level in *S. aureus*-infected RAW264.7 cells; moreover, propofol resulted in an increase in bacterial survival by inhibiting ROS and phagocytosis.

## Introduction

Intravenous anesthetics may be used for the sedation of perioperative and critically ill patients but also have potential immunomodulatory effects [1–3]. Intravenous anesthetics can inhibit human phagocyte functions, including chemotaxis, cytokine secretion, phagocytosis, and reactive oxygen species (ROS) generation [1, 4–8]. The disruption of any phagocyte function could result in bacterial infections.

*Staphylococcus aureus* is one of the most important pathogens that cause human morbidity and mortality worldwide [9]. *S. aureus* can lead to various infections ranging from minor skin and soft tissue infections to life-threatening invasive diseases, such as necrotizing pneumonia and sepsis [9–11]. *S. aureus* acts via several mechanisms to escape host immune defenses and establish an infection, including resisting antimicrobial peptides, clearing ROS, inhibiting complement activation and neutrophil recruitment, and evading phagocytosis [12, 13].

Among host defense mechanisms, IL-1β secreted by phagocytes is important in protecting against *S. aureus* infections. IL-1β-deficient mice have demonstrated the importance of IL-1β in neutrophil infiltration and deep *S. aureus* skin infections [14, 15]. Two major steps are involved in IL-1β secretion. First, the expression of pro-IL-1β is induced by microbial components. Second, the IL-1β precursor (34 kDa) is cleaved into its mature form (17 kDa) by inflammasome-activated caspase 1 or released through an unknown caspase 1-independent pathway [16]. Previous papers have shown that propofol can decrease IL-1β secretion in macrophages [17] and that several general anesthetics inhibit IL-1β mRNA expression in LPS-stimulated glial cells and primary microglia [18, 19]. However, the regulatory mechanisms of intravenous anesthetics regarding IL-1β secretion remain unclear.

Although intravenous anesthetics may have immunomodulatory effects, the literature is controversial. For example, propofol has been shown to significantly reduce chemotaxis, phagocytosis, and ROS production in macrophages [7, 8], but these results are not consistent with the studies from Ploppa et al. [20, 21]. The use of different assay systems might contribute to these discrepancies. Therefore, we used one system, *S. aureus*-infected RAW264.7 cells, to compare the effects of four intravenous anesthetics on IL-1β secretion, ROS levels, and bacterial survival.

## Materials and methods

### Bacteria, cell culture, and bacterial infection

*S. aureus* ATCC 25923 was grown on blood agar (tryptic soy agar with 5% sheep blood) and sub-cultured in Luria–Bertani (LB) broth for 16 h for the infection assay. The mouse macrophage cell line RAW264.7 was purchased from the Food Industry Research and Development Institute in Taiwan. The cells were grown in DMEM (Gibco BRL, Grand Island, NY, USA) containing 10% FBS and were sub-cultured every 2–3 days. To conduct the *S. aureus* infection assays, a 10-fold dilution of bacteria (OD 600 = 1) was added to RAW264.7 cells for a 30-min incubation. Next, free bacteria were washed away with 1× PBS, and a multiplicity of infection (MOI) of 15 was established; DMEM supplemented with 2% FBS was then added for incubation and further analyses.

### Reagents

Propofol lipid emulsion, thiamylal sodium, midazolam, and ketamine were purchased from Fresenius Kabi Austria GmbH (Graz, Austria), Shinlin Sinseng Pharmaceutical Co., Ltd. (Taoyuan, Taiwan), Nang Kuang (Tainan, Taiwan), and Taiwan Pfizer Inc. (New Taipei, Taiwan), respectively. The NADPH oxidase inhibitor diphenyleneiodonium chloride (DPI) purchased from Abcam (ab141310, Cambridge, UK) was used to inhibit ROS production.

### Cytotoxicity assay

The lactate dehydrogenase (LDH) activity present in lysed RAW264.7 cells infected with *S. aureus* and treated with or without intravenous anesthetics was determined using a Cytotoxicity Detection Kit^PLUS^ (Roche Applied Science, Mannheim, Germany), according to the manufacturer’s instructions.

### Viability assay

Cell counting kit-8 (Sigma-Aldrich, Inc., St. Louis, MO, USA) assays were used to evaluate cell viability. The adjusted concentrations of intravenous anesthetics used for the cell viability assay were based on the clinically relevant concentrations of propofol (6 µg/ml), thiamylal sodium (30 µg/ml), midazolam (1.5 µg/ml), and ketamine (2 µg/ml); these concentrations are in accordance with the typical therapeutic doses given by intravenous injection to adult patients. RAW264.7 cells (1 × 10^5^ cells/well) suspended in DMEM with 10% FBS were seeded in a 96-well microplate and incubated overnight. After adherence, the cells were treated with one, two, or five times the clinically relevant concentrations of intravenous anesthetics for 5 h and were then washed 3 times with 1× PBS. Then, cell medium containing a WST-8 solution was added for up to 2 h to measure the metabolically active cells. Finally, cell viability was measured using a Model 680 microplate reader (absorbance at 450 nm; reference at 655 nm) (Bio-Rad Laboratories, Inc., Hercules, CA, USA). The cell viability was calculated using the following formula: *A*_treated_/*A*_control_.

### Intravenous anesthetic treatment of *S. aureus*-infected RAW264.7 cells

RAW264.7 cells were preincubated with the intravenous anesthetics for 30 min and then infected with *S. aureus* suspended in medium containing the same concentrations of intravenous anesthetics for 30 min. After the incubation period, the cells were washed with 1× PBS, and the same concentrations of intravenous anesthetics were added again; the time of this addition served as the starting time point (0 h). RAW264.7 cells and *S. aureus*-infected RAW264.7 cells treated without intravenous anesthetic served as the negative and positive controls, respectively. For this infection model, IL-1β mRNA and protein expression levels were determined using cell pellets collected at 3 h from 6-well plates (1 × 10^6^ cells/well), whereas IL-1β secretion was assayed using supernatants sampled at 4 h from a 96-well microplate (1 × 10^5^ cells/well); these supernatants were assayed using a mouse IL-1β ELISA kit (Elabscience, Houston, Texas, USA), according to the manufacturer’s instructions.

### Quantitative polymerase chain reaction

Total RNA was isolated using Trizol reagent (Invitrogen Corporation, Carlsbad, CA, USA) according to the manufacturer’s procedures. The concentration and integrity of the total RNA were evaluated based on the A260/280 ratio. mRNA levels were quantified using the SuperScript™ III Platinum™ One-Step qRT-PCR Kit (Thermo Fisher Scientific Inc., Carlsbad, CA, USA) and the TaqMan probes and primers for GAPDH (Mm99999915_g1) and customized IL-1β probes and primers (primers: forward: 5′GTCCTGATGAGAGCATCCAG3′, reverse: 5′CGGGAAAGACACAGGTAGC3′; probe: TCGCAGCAGCACATCAACAAGAGC). Quantitative polymerase chain reaction (qPCR) assays were performed using the CFX96™ Real-Time PCR Detection System (Bio-Rad Laboratories, Inc., Foster, CA, USA). IL-1β gene expression was normalized to GAPDH and shown as the fold change relative to the control cells.

### Western blot analysis

Cells were lysed with M-PER mammalian protein extraction reagent (Thermo Scientific Inc., Rockford, IL, USA) containing 0.1% protease inhibitor cocktail. Equal amounts (40 µg) of samples were run on SDS-PAGE gels and transferred to PVDF membranes. The membranes were then incubated with anti-mouse IL-1β/IL-1F2 (R&D Systems, Inc., Minneapolis, MN, USA) and anti-GAPDH (GeneTex, Inc., Irvine, CA, USA) antibodies, followed by incubation with secondary antibodies conjugated to horseradish peroxidase (HRP). After incubation with Immobilon Western Chemiluminescent HRP substrate (EMD Millipore Corporation, Billerica, MA, USA), the signals were detected using the BioSpectrum Imaging System (UVP).

### Luminol chemiluminescence assay

Luminol (5-amino-2,3-dihydro-1,4-phthalazinedione) freely permeates through the cell membrane and was used to detect the total amount of intracellular and extracellular ROS produced by NADPH oxidase. RAW264.7 cells (1 × 10^5^ cells/well) were preincubated with intravenous anesthetics in a 96-well white microplate for 30 min; then, *S. aureus* with the same concentrations of intravenous anesthetics were added to the cells for 30 min. After washing twice with 1× PBS, the cells were stained with 0.05 mg/ml luminol (Sigma-Aldrich, Inc., St. Louis, MO, USA). The increase in the chemiluminescence resulting from ROS production was measured immediately and was analyzed continuously over 1 h at 37 °C using a BioTek Synergy H1 microplate reader (BioTek, Winooski, VT, USA). RAW264.7 cells that were not preincubated with intravenous anesthetics but with *S. aureus* incubation were used as a positive control. Treatment with each intravenous anesthetic alone was also used as a control. The peak value of chemiluminescence at 5 min was used to calculate the relative effect of the intravenous anesthetics on ROS production.

### Bacterial survival assay

Bacterial survival assays were conducted on RAW264.7 cells that had undergone intravenous anesthetic pretreatment and were then infected with *S. aureus* and incubated with intravenous anesthetics. Moreover, cells infected with *S. aureus* but without intravenous anesthetic treatment were used as a reference control. Each infection condition occupied two wells, allowing the bacteria to be counted at two time points (0 and 3 h). The first well contained cells that were infected with *S. aureus* for 30 min and then washed with 1× PBS (0 h). The second well had the same conditions as the first well, but the cells were further incubated for 3 h. Finally, the cell suspensions were serially diluted, and these dilutions were plated on LB agar plates. Colony forming units (CFU) were counted the following day, and changes in bacterial survival were calculated as the CFU at 3/0 h.

### Phagocytosis of *S. aureus* bioparticles

Phagocytosis was assessed using pHrodo™ Green *S. aureus* Bioparticles^®^ Conjugate (P35367, Life technologies, Carlsbad, CA, USA), according to the manufacturer’s instructions. These *S. aureus* particles were conjugated to pHrodo™ dye, which dramatically increases green fluorescence at an acidic pH, and were suitable for quantitative measurements of phagocytosis. RAW264.7 cells were suspended in Opti-MEM^®^, seeded in a 96-well plate (1 × 10^5^ cells/well) and incubated overnight. pHrodo *S. aureus* bioparticles conjugates were resuspended in Live Cell Imaging Solution (1 mg/ml). The cells in experimental wells were pretreated with propofol or cytochalasin D for 30 min, and then, pHrodo *S. aureus* particles combined with or without propofol were added to the cells. The positive control was the cells treated only with *S. aureus* particles without pretreatment. The plates were covered and incubated at 37 °C for 2 h in the absence of CO_2_. Fluorescence was recorded using a BioTek Synergy H1 microplate reader (BioTek, Winooski, VT, USA) with 490-nm excitation and 540-nm emission wavelengths. Additionally, the cells that had been incubated with *S. aureus* particles were stained with Hoechst 33342 or LysoTracker Red DND-99 (Invitrogen Life Technologies, CA, USA) to label acidified compartments for live cell imaging. Fluorescence microscopy (Olympus Corporation, Tokyo, Japan) was used to visually investigate phagocytosis.

## Results

### *S. aureus* induced IL-1β secretion in RAW264.7 cells

**Fig. 1:**
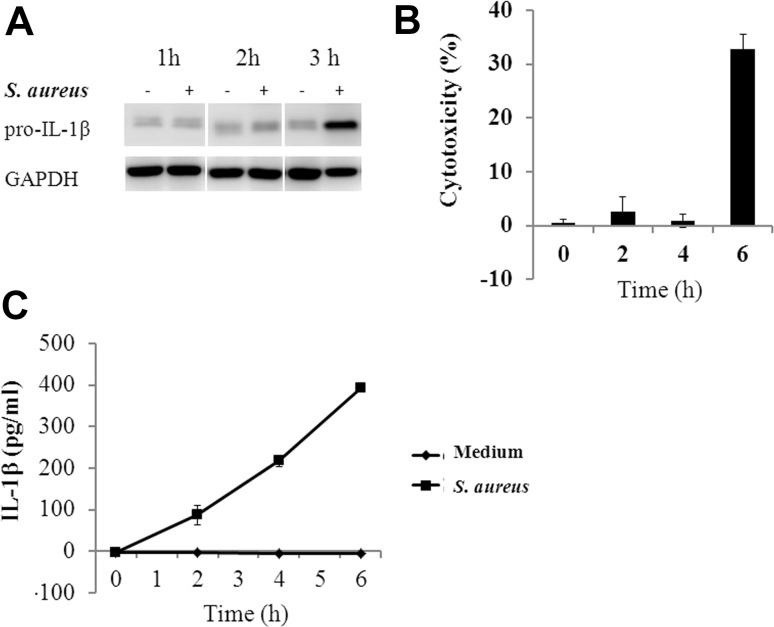
Kinetics of the production and secretion of IL-1β in *S. aureus*-infected RAW264.7 cells. RAW264.7 cells were co-cultured with *S. aureus* for different periods of time. **a** Western blot of intracellular pro-IL-1β at the indicated times. **b** IL-1β secretion at 2, 4, and 6 h was measured by ELISA. **c** Cell cytotoxicity was measured by LDH release and is shown as a percentage relative to the total cell lysis after Triton X-100 detergent treatment. The data obtained from three individual experiments are expressed as the means ± SD

*Staphylococcus aureus* slightly induced intracellular pro-IL-1β at 2 h, and these levels increased dramatically at 3 h (Fig. 1a). In the supernatant of this infection condition, secreted IL-1β was detectable at 2 h and increased in a time-dependent manner (Fig. 1b). To exclude IL-1β secretion due to cell lysis, we measured LDH activity at different time points. A high percentage (32.8%) of cell death occurred at 6 h (Fig. 1c). Therefore, the maximum time for sample collection was set at 4 h.

### Intravenous anesthetics decreased IL-1β secretion

**Fig. 2:**
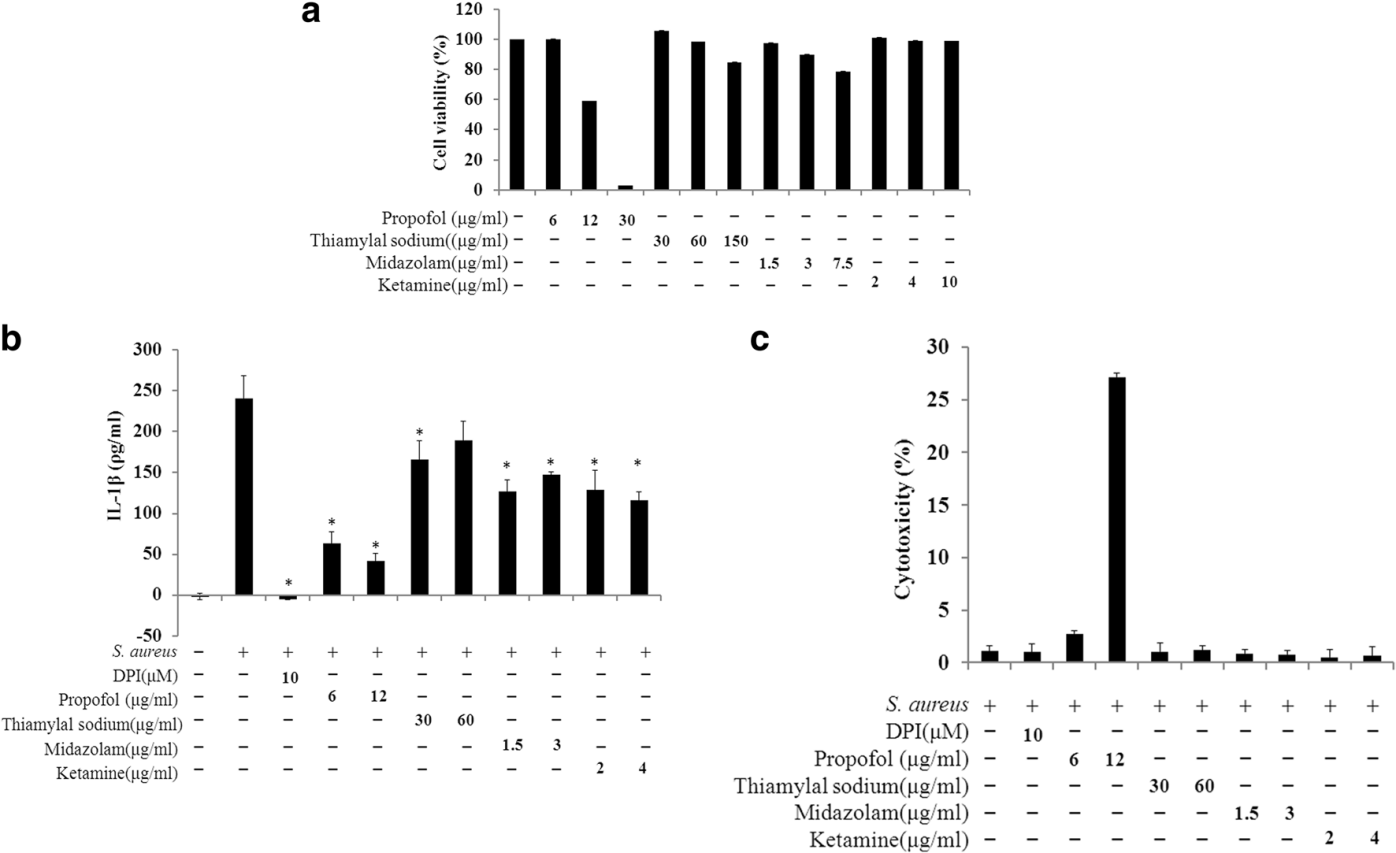
Cell viability and inhibition of IL-1β secretion in RAW264.7 cells treated with intravenous anesthetics. **a** Cell viability after treatment with a range of concentrations of the intravenous anesthetics (1, 2, and 5 times the clinically relevant concentrations) was assessed using a CCK-8 assay. **b** Intravenous anesthetics (1 and 2 times the clinically relevant concentrations) were added prior to and during the infection with *S. aureus*, except for the control cells and the *S. aureus*-infected positive control. After 4 h of infection, the IL-1β levels in the cell culture supernatants were analyzed using an ELISA. **c** Cytotoxicity after *S. aureus* infection and treatment with intravenous anesthetics was detected using an LDH assay. The data obtained from three individual experiments are presented as the means ± SD. *p < 0.05 compared to the *S. aureus*-infected positive control according to the two-tailed Mann–Whitney test

Cell viability after treatment with one, two, and five times the clinically relevant concentrations of intravenous anesthetics was investigated to avoid cytotoxicity in RAW264.7 cells. Propofol (12 and 30 µg/ml) treatment decreased cell viability by > 40%. However, the viability remained as high as 90% when the cells were treated with one or two times the clinically relevant concentrations of the other three intravenous anesthetics (Fig. 2a). Next, we investigated whether intravenous anesthetics decrease IL-1β secretion in RAW264.7 cells infected with *S. aureus*. The lowest level of secreted IL-1β was detected with propofol treatment of the *S. aureus*-infected RAW264.7 cells (Fig. 2b). Secreted IL-1β also decreased following treatment with thiamylal sodium, midazolam, and ketamine, but treatment at higher concentrations (two times the clinically relevant concentrations) did not further decrease the IL-1β secretion. LDH activity was also examined to confirm that cell death was rare for each infection condition; this was the case for each infection condition except the treatment with 12 µg/ml propofol (Fig. 2c). The NADPH oxidase inhibitor DPI blocked IL-1β secretion (Fig. 2b).

### Thiamylal sodium and midazolam inhibited IL-1β mRNA and pro-IL-1β synthesis

**Fig. 3:**
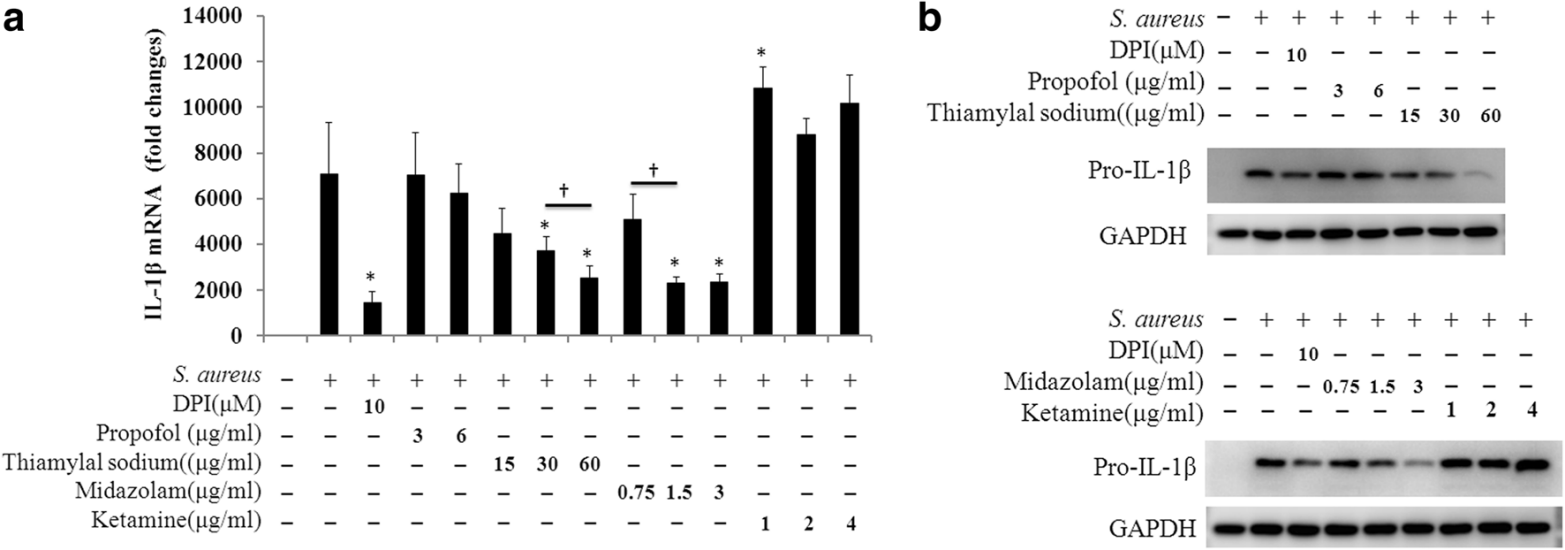
IL-1β mRNA and pro-IL-1β biosynthesis modulation by intravenous anesthetics. RAW264.7 cells (1 × 10^6^/well) were exposed to media only or *S. aureus* in the absence or presence of intravenous anesthetics for 3 h. **a** IL-1β mRNA was detected by a qPCR assay. The data are presented as the fold change versus the control cells; the data were obtained from three individual experiments. * indicates a significant difference compared to the *S. aureus*-infected positive control using the two-tailed Mann–Whitney test (p < 0.05). † indicates a significant difference between the two marked groups. **b** Pro-IL-1β biosynthesis was measured via western blot analysis. One set of representative data obtained from three individual experiments is presented. The experiments were performed using 0.5, 1, and 2 times the clinically relevant concentrations of the anesthetics, but propofol was used at only 0.5 and 1 times its clinically relevant concentration

We next determined whether intravenous anesthetics decreased the expression of IL-1β mRNA and protein; thiamylal sodium and midazolam treatment inhibited IL-1β mRNA and pro-IL-1β expression in a dose-dependent manner (Fig. 3a, b). In contrast, propofol did not cause similar effects, and ketamine increased IL-1β mRNA and pro-IL-1β expression (Fig. 3a, b). DPI strongly inhibited IL-1β mRNA (80%) and pro-IL-1β production.

### *S. aureus*-induced ROS production and bacterial survival were modulated by intravenous anesthetics

**Fig. 4:**
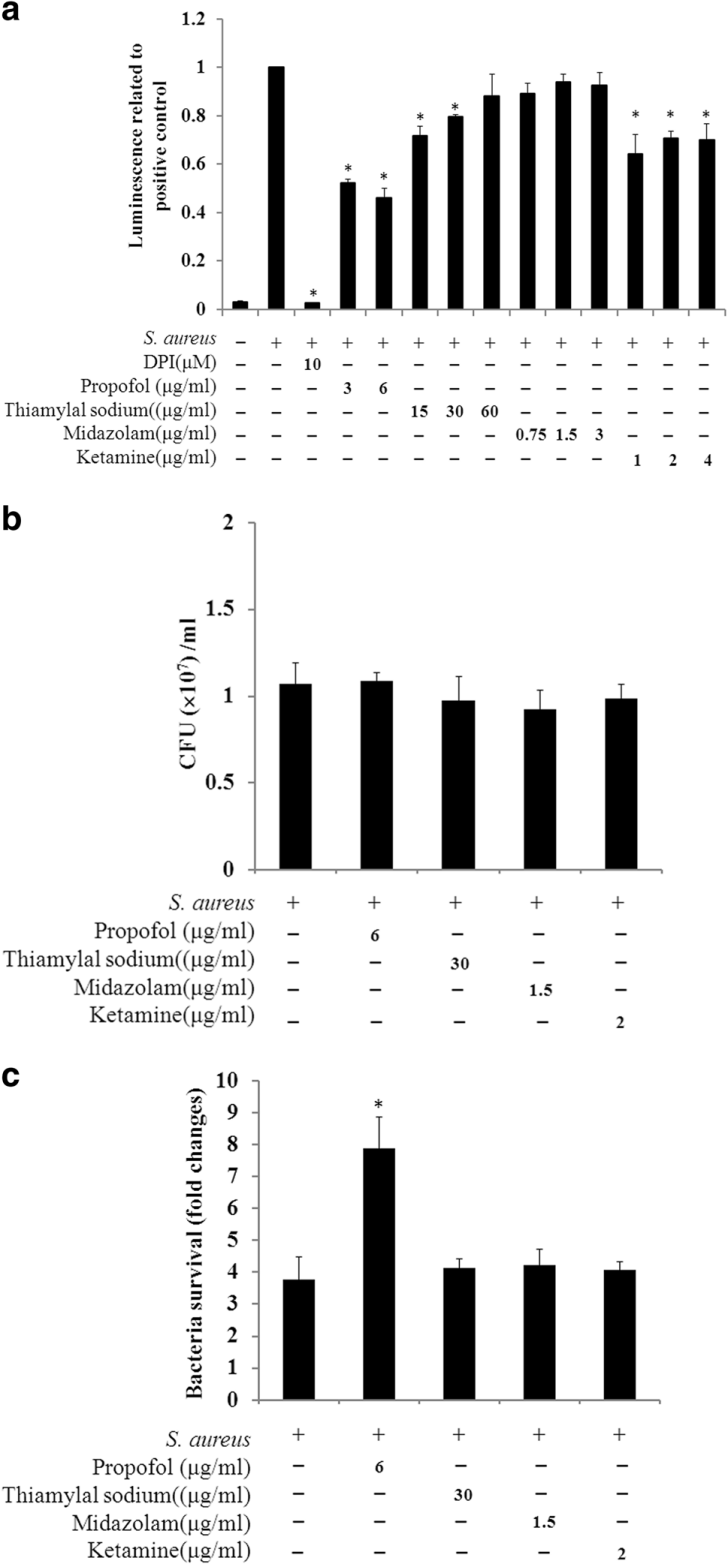
Effect of intravenous anesthetics on ROS level and bacterial survival. **a** Intravenous anesthetics suppressed *S. aureus*-induced ROS. RAW264.7 cells were incubated in the absence or presence of intravenous anesthetics prior to and during the infection with *S. aureus*. Treatment with 10 µM DPI was used to inhibit ROS as a control. For the positive control, the maximum luminescence produced upon *S. aureus* infection was used as the reference value. The relative ratio was calculated from the luminescence value induced by co-incubation with a drug and the bacteria subtracted from the drug control and then divided by the luminescence of the positive control. A range of concentrations (0.5, 1, and 2 times the clinically relevant concentrations) of intravenous anesthetics was used, except for propofol (for which 0.5 and 1 times the clinically relevant concentration were used). The data are presented as the means ± SD of three replicates. **b** Similar cell-associated bacterial numbers with different intravenous anesthetic treatments. After incubation with *S. aureus* without or with intravenous anesthetics for 30 min, RAW264.7 cells were washed and then suspended for growth and counting of the cell-associated colony forming units (CFUs) that started the infection (0 h). Intravenous anesthetics were used at their clinically relevant concentrations. **c** Propofol treatment increased *S. aureus* survival. Bacterial CFUs were grown and counted at the end of infection (3 h). Fold changes in bacterial survival were calculated based on the bacterial number at 3 h divided by the bacterial number at 0 h. The data are presented as the means ± SD of three individual experiments. Significance was analyzed using the two-tailed Mann–Whitney test (p < 0.05). * indicates a significant difference compared with the *S. aureus*-infected control

**Fig. 5:**
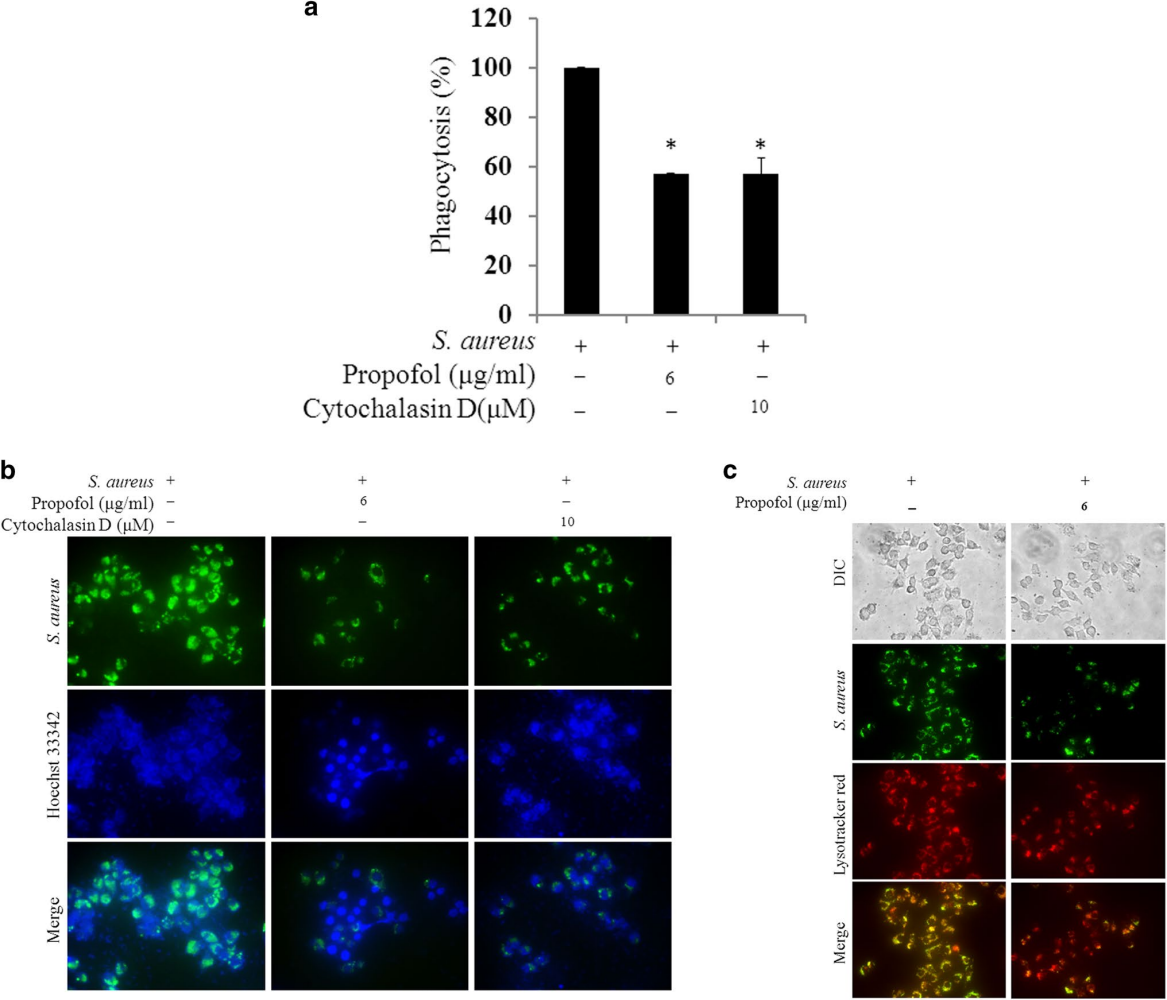
Propofol inhibited phagocytosis of RAW264.7 cells. **a** RAW264.7 cells pretreated with propofol were further co-incubated with pHrodo™ Green *S. aureus* Bioparticles^®^ Conjugates and propofol (6 µg/ml). Net phagocytosis was calculated by subtracting the average fluorescence intensity of the no-cell negative-control wells from that of the positive control, propofol, and cytochalasin D (phagocytosis inhibitor) treatment wells. The phagocytic ability related to the positive control was calculated. The data are presented as the means ± SD of three individual experiments. Significance was analyzed using the two-tailed Mann–Whitney test (p < 0.05). * indicates a significant difference compared with the *S. aureus* bioparticles (positive control). **b** Live cell imaging of phagocytosis. After the RAW264.7 cells were incubated with green *S. aureus* bioparticles, the nuclei were stained with Hoechst 33342 (blue). **c** Localization of phagocytosis and acidified compartments. LysoTracker Red DND-99 (50 nM) was used to label acidified compartments for 10 min. The images were investigated using a 40× objective

In the *S. aureus*-infected RAW264.7 cells, ROS level was significantly inhibited by three of the intravenous anesthetics: propofol, thiamylal sodium, and ketamine (Fig. 4a). Of these treatments, propofol yielded the lowest ROS level, and the clinically relevant concentration of propofol reduced ROS level by > 50%. To confirm *S. aureus* survival during infection along with intravenous anesthetic treatment, we assessed the cell-associated bacterial number after 30 min of infection as the starting time point. At this time point, similar bacterial numbers were found for RAW264.7 cells receiving the different intravenous anesthetic treatments (Fig. 4b). Three hours later, the bacterial number was significantly increased in *S. aureus*-infected RAW264.7 cells that received propofol treatment. The other intravenous anesthetics did not affect bacterial survival compared with the untreated *S. aureus*-infected cells (Fig. 4c). We further examined whether propofol promoted bacterial survival by minimizing the phagocytic ability and found that propofol actually inhibited the phagocytosis of *S. aureus* BioParticles (Fig. 5a). The amount of green *S. aureus* was substantially decreased in the RAW264.7 cells with propofol treatment, similar to the results with the anti-phagocytosis effector cytochalasin D (Fig. 5b). *S. aureus* bioparticles exhibited colocalization with the LysoTracker label in RAW264.7 cells with or without propofol treatment (Fig. 5c).

## Discussion

*Staphylococcus aureus* infections cause various clinical pathological changes, from skin infection to severe sepsis, and macrophages play important roles in controlling these infections. Previous papers have demonstrated that anesthetics can inhibit several functions of phagocytes, including chemotaxis, phagocytosis, and ROS generation [1, 4–8]. Comparing the effects of different anesthetics on immunomodulation could result in information beneficial for choosing a clinical anesthetic. In this study, we used a model of *S. aureus*-infected RAW264.7 cells and found that of four intravenous anesthetics, propofol was the most effective agent for inhibiting IL-1β secretion, but this anesthetic did not decrease IL-1β mRNA or pro-IL-1β biosynthesis. Although thiamylal sodium and midazolam caused a dose-dependent decrease in IL-1β mRNA and pro-IL-1β biosynthesis, the suppression of IL-1β secretion by these agents was modest. Propofol also considerably reduced *S. aureus*-induced ROS production, and compared with *S. aureus*-infected RAW264.7 cells alone or with treatment with the other intravenous anesthetics, only propofol resulted in increased bacterial survival.

Chen et al. [17] demonstrated that RAW264.7 cells released the highest level of IL-1β (50 pg/ml) after LPS stimulation for 6 h. In this study, we used live *S. aureus* to cause an acute infection, and the RAW264.7 cells secreted up to 250 pg/ml of IL-1β at 4 h. These results indicate that RAW264.7 cells are sensitive to *S. aureus* infection and that this model can be used to study IL-1β secretion. Live bacteria can also cause direct interactions between bacteria and cells.

Previous papers have revealed that propofol can reduce superoxide generation and elastase release in fMLF-activated human neutrophils, as well as polymorphonuclear leukocyte (PMNL) chemotaxis and cytokine secretion in LPS-activated macrophages [1, 17, 22]. In Fig. 2b, propofol considerably reduced IL-1β secretion. This result is consistent with a previous study using LPS-activated macrophages [17]. Studies related to the effects of the other intravenous anesthetics on macrophage-secreted IL-1β are limited, but a few studies have shown that midazolam induced significant decreases in IL-1β levels in the blood of critically ill surgical patients [3] and that ketamine significantly inhibited IL-1β mRNA in LPS-stimulated macrophages [6]. When comparing the levels of IL-1β secretion in the *S. aureus*-stimulated macrophages treated with the four intravenous anesthetics, we found that non-cytotoxic levels of propofol (6 µg/ml) suppressed IL-1β secretion most strongly (Fig. 2b), even though the other intravenous anesthetics were used at 2 times their clinically relevant concentrations. All of the data indicate that these four intravenous anesthetics have the potential to inhibit IL-1β secretion, particularly propofol.

Previous studies have shown that propofol and ketamine can decrease IL-1β mRNA levels in LPS-activated macrophages [6, 17]. In contrast, in this study, IL-1β mRNA in macrophages exposed to *S. aureus* was not reduced by propofol but was increased by ketamine treatment (Fig. 3a); these results are consistent with IL-1β biosynthesis (Fig. 3b). The discrepancy between previous studies and our study may be due to the diverse stimulants (LPS and *S. aureus*) and the different mechanisms regulating IL-1β mRNA. Additionally, exposure to thiamylal sodium and midazolam inhibited IL-1β mRNA and pro-IL-1β biosynthesis in a dose-dependent manner (Fig. 3a, b), but the suppression of IL-1β secretion by these agents was modest (Fig. 2b). Moreover, DPI treatment induced less pro-IL-1β synthesis but completely blocked IL-1β secretion (Figs. 3b, 2b). These results support the conclusion that posttranslational regulation is the critical step for controlling IL-1β secretion [23]. We also hypothesized that propofol and ketamine decreased IL-1β secretion via this mechanism because these two anesthetics did not decrease pro-IL-1β mRNA or protein biosynthesis (Fig. 3a, b).

In this study, the administration of DPI (an ROS inhibitor) to *S. aureus*-infected macrophages resulted in decreased IL-1β mRNA and pro-IL-1β synthesis (Fig. 3a, b); these results confirmed those of Tassi et al. [23]. ROS can induce NF-κB activation; NF-κB is one of the factors required for the transcription of proinflammatory cytokines, including IL-1β [24, 25]. Propofol, midazolam, and ketamine have been demonstrated to suppress ROS production in phagocytes [1, 6, 26]. Of the intravenous anesthetics used in this study, propofol was the best at reducing *S. aureus*-induced ROS levels (Fig. 4a). However, propofol did not affect IL-1β transcription (Fig. 3a). The effects of thiamylal sodium and midazolam on suppressing ROS levels were less than those of propofol, but thiamylal sodium and midazolam significantly inhibited IL-1β mRNA expression (Fig. 3a). These results suggest that ROS was not the only determinant factor in the regulation of IL-1β mRNA in our assay system.

Bacterial survival was particularly increased when the *S. aureus*-infected macrophages were treated with propofol (Fig. 4c). Propofol did not influence the cell-associated bacterial number at the beginning of infection (Fig. 4b) and bacterial growth (data not shown). Phagocytosis and ROS are critical for controlling bacterial infections. In our study, propofol significantly inhibited the ROS level and phagocytic ability (Figs. 4a, 5a, b) but not the formation of acidified compartments (Fig. 5c). These results provide direct evidence for the anti-phagocytic effects of propofol on *S. aureus*, which indicate that propofol contributes to the increase in bacterial survival due to the inhibition of ROS and phagocytosis.

## Conclusions

**Fig. 6:**
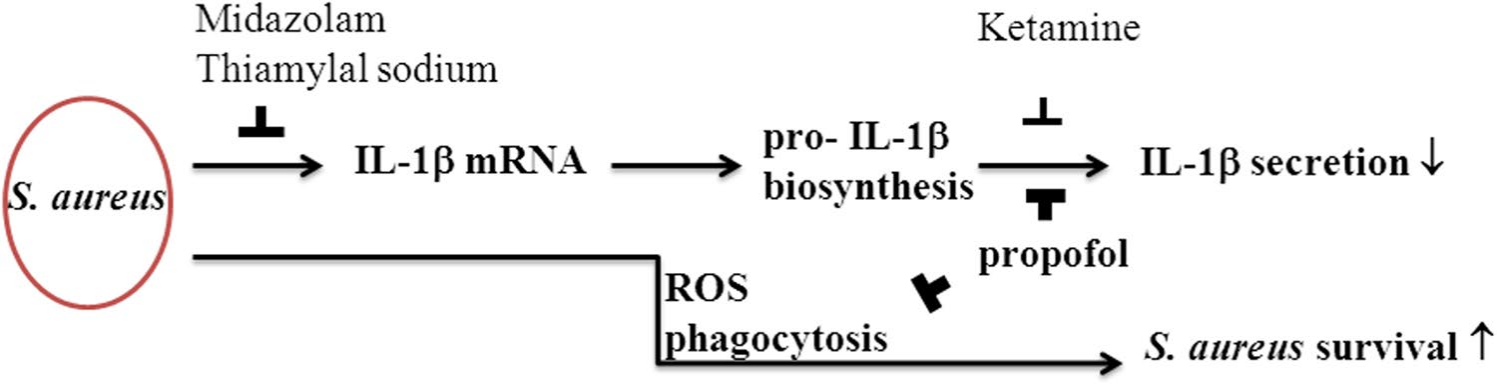
The main effects of four intravenous anesthetics on IL-1β secretion and *S. aureus* survival. Midazolam and thiamylal sodium primarily inhibited pro-IL-1β mRNA and biosynthesis. Propofol and ketamine resulted in a decrease in IL-1β secretion. Moreover, propofol particularly increased *S. aureus* survival by suppressing ROS and phagocytosis

Among the four intravenous anesthetics, thiamylal sodium and midazolam inhibited pro-IL-1β mRNA in a dose-dependent manner in the *S. aureus-*infected RAW264.7 cells; however, propofol was the most effective at inhibiting the macrophage functions of IL-1β secretion and ROS level and did not decrease pro-IL-1β biosynthesis. Moreover, propofol increased bacterial survival though the suppression of ROS and phagocytosis (Fig. 6).
